# Palliative care interventions in intensive care unit patients – a systematic review protocol

**DOI:** 10.1186/s13643-019-1064-y

**Published:** 2019-06-22

**Authors:** Victoria Metaxa, Despina Anagnostou, Savvas Vlachos, Nishkantha Arulkumaran, Ingeborg van Dusseldorp, Sherihane Bensemmane, Rebecca Aslakson, Judy E. Davidson, Rik Gerritsen, Christiane Hartog, Randall Curtis

**Affiliations:** 1 King’s College Hospital, London, SE5 9RS UK; 2 Human Health Sciences, School of Medicine, Kyoto University, Kyoto, Japan; 3 Bloomsbury Institute of Intensive Care Medicine, University College London, London, UK; 4 Medical Information Specialist, Medical Centre Leeuwarden, MCL Academy, Leeuwarden, Netherlands; 5 European Society of Intensive Care Medicine, Rue Belliard 19, 1040 Bruxelles, Belgium; 6 Department of Medicine, Division of Primary Care and Population Health, Palliative Care Section, Stanford University, Stanford, CA 94305 USA; 7 Department of Anesthesiology, Perioperative, and Pain Medicine, Stanford University, Stanford, CA 94305 USA; 8 Department of Nursing, University of California San Diego Health, San Diego, USA; 9 Centrum voor Intensive care, Medisch Centrum Leeuwarden, PO Box 888, 8901BR Leeuwarden, Netherlands; 10 Department for Anesthesiology and Intensive Care, Jena University Hospital, Jena, Germany; 11 Cambia Palliative Care Center of Excellence at UW Medicine, Harborview Medical Center, University of Washington, 325 Ninth Avenue, Box 359762, Seattle, WA 98104 USA; 12 Ethics Section, European Society of Intensive Care Medicine (ESICM), Brussels, Belgium; 13 Systematic Reviews Group, European Society of Intensive Care Medicine (ESICM), Brussels, Belgium

**Keywords:** Palliative care, Intensive care, End-of-life care

## Abstract

**Background:**

Even though data suggest that palliative care (PC) improves patient quality of life, caregiver burden, cost, and intensive care unit (ICU) length of stay, integration of PC in the ICU is far from being universally accepted. Poor understanding of what PC provides is one of the barriers to the widespread implementation of their services in ICU. Evidence suggests that the availability of specialist PC is lacking in most European countries and provided differently depending on geographical location. The aim of this systematic review is to compare the numbers and types of PC interventions and gauge their impact on stakeholder outcomes and ICU resource utilisation.

**Methods:**

We will undertake a systematic review of the published peer-reviewed journal articles; our search will be carried out MEDLINE, Embase, Cochrane, CINAHL, and PsycINFO. The search strategy will include variations in the term ‘palliative care’ and ‘intensive care’. All studies with patient populations undergoing palliative care interventions will be selected. Only full-text articles will be considered, and conference abstracts excluded. There will be no date restrictions on the year of publications or on language. The primary aim of the present study is to compare the numbers and types of PC interventions in ICU and their impact on stakeholder (patient, family, clinician, other) outcomes. Reporting of findings will follow the Preferred Reporting Items of Systematic Reviews and Meta-Analyses (PRISMA) guidelines.

**Discussion:**

This review will provide insight into the implementation of palliative care in ICU, elucidate differences between countries and health systems, reveal most effective models, and contribute to identifying research priorities to improve outcomes.

**Systematic review registration:**

International Prospective Register of Systematic reviews PROSPERO (CRD42018094315)

## Background

Palliative care (PC) is a holistic approach which incorporates management of physical, psychological and spiritual symptoms, communication regarding goals of care, support for clinicians and families, and planning for care transitions in patients with critical illness [1]. Even though data suggest that PC improves quality of life, and decreases caregiver burden, cost [2, 3], and hospital and intensive care unit (ICU) length of stay [4], the integration of PC in the ICU is not universally accepted. Poor understanding of what PC provides is one of the barriers to the widespread implementation of their services [5] in ICU. Evidence suggests that specialist PC is lacking from most European countries and provided differently depending on geographical location [6]. This is further complicated by the fact that end-of-life (EoL) practices also vary between countries and healthcare systems. For instance, EoL care is more hospital-centric in the USA than in Europe, although ICU admissions in the last days of life are more prevalent in Europe compared to the USA [7]. Three separate models have been proposed to aid better PC-ICU integration: improving palliative care by ICU clinicians, as part of routine ICU practice (integrative model); improving palliative care by utilising specialist PC teams (consultative model); and a mixed model that incorporates both of these strategies.

Recently, existing evidence was organised to address: (1) opportunities to alleviate physical and emotional symptoms, improve communication, and provide support for patients and families; (2) models and specific interventions for improving ICU palliative care; (3) available resources for ICU palliative care improvement; and (4) ongoing challenges and targets for future research [8]. However, the methods and specific tools used to assess the impact of PC in ICU are varied [9, 10]. Furthermore, previous studies in PC have used heterogeneous outcomes that often fail to capture the multi-dimensional nature of the discipline and the varied needs of different stakeholders [4]. The most effective and efficient way to provide PC in ICU is largely unknown [8].

The aim of this systematic review was to compare the numbers and types of PC interventions and gauge their impact on stakeholder outcomes and ICU resource utilisation.

## Objectives

The primary aim of this study is to compare the numbers and types of PC interventions in ICU, and their impact on patient and family outcomes.

Secondary aims are to

- Assess the models for integrating palliative care into the ICU
- Compare evidence from different regions of the world (Europe, North America, South America, Asia).

This will enable us to identify research priorities and formulate a research agenda to improve palliative care in ICU.

## Methods

In order to assess the current state of evidence, we propose to describe the numbers and types of PC interventions and gauge their impact on stakeholder (patients, families, and healthcare providers) outcomes and ICU resource utilisation. We also intend to assess the different types of PC and their variable integration in European versus non-European countries and provide data to develop future interventions to improve PC in the ICU.

### Data sharing

This systematic review protocol is registered with the PROSPERO International Prospective Register of Systematic Reviews (https://www.crd.york.ac.uk/prospero/display_record.php?RecordID=94315) (CRD42018094315) and will be reported using Preferred Reporting Items of Systematic Reviews and Meta-Analyses (PRISMA) guidelines in the peer-reviewed literature [11].

### PICO question

We have undertaken a systematic review of the published peer-reviewed journal articles. We have defined the PICOS criteria as follows:

#### Population

Adult patients (> 18 years of age) admitted to the intensive care unit or high-dependency areas.

Terms used:

(burn unit*[tiab] OR coronary care unit*[tiab] OR respiratory care unit*[tiab] OR intensive cardiac care [tiab] OR “Intensive Care Units”[Mesh:NoExp] OR icu [tiab] OR high dependency unit [tiab] OR hdu [tiab]) OR “Burn Units”[Mesh] OR “Coronary Care Units”[Mesh] OR “Respiratory Care Units”[Mesh].

#### Intervention

Palliative care interventions occurring in ICU.

Terms used:

(“Palliative Care”[Mesh] OR “Palliative Medicine”[Mesh] OR “Hospice and Palliative Care Nursing”[Mesh] OR “Terminal Care”[Mesh] OR “Hospice Care”[Mesh] OR palliat*[tiab] OR eol care [tiab] OR EOLC [tiab] OR terminal care [tiab] OR end of life [tiab] OR terminal illness [tiab] OR terminal patient*[tiab] OR terminally ill [tiab] OR limited survival [tiab] OR critically ill [tiab] OR terminal phase [tiab] OR terminal stage [tiab] OR life-limiting [tiab] OR bereavement care [tiab] OR bereavement care [tiab] OR bereavement counselling [tiab] OR comfort care [tiab] OR symptom management [tiab] OR symptomatic treatment [tiab] OR symptomatic therapy [tiab] OR hospice program*[tiab] OR hospice care [tiab] OR limited life [tiab] OR supportive care [tiab] OR supportive need*[tiab] OR supportive treatment [tiab] OR supportive therapy [tiab])

#### Comparator

Patients that did not receive any PC interventions.

Terms used: none.

#### Outcome

The number and type of PC interventions as identified by a working group funded by the Robert Wood Johnson Foundation (patient- and family-centred care, communication, continuity of care, emotional and practical support of patients and families, symptom management and comfort care, spiritual support, and emotional and organisational support for ICU clinicians) in ICU in Europe, North America, South America, and Asia [12]. We will also assess the effect of these interventions on the domains of patient/family-, clinician-, systems-, content-related outcomes, evaluating the different PC models.

Terms used: none.

#### Studies

Controlled trials (randomised and non-randomised). Case reports, case series, editorials/commentaries, opinion papers, studies with no outcome data, small studies (< 30 patients), publications only as abstracts, and (non-systematic) review papers will not be included.

Terms used:

((randomised controlled trial [pt] OR controlled clinical trial [pt] OR randomised controlled trials [mh] OR random allocation [mh] OR double-blind method [mh] OR single-blind method [mh] OR clinical trial [pt] OR clinical trials [mh] OR “clinical trial”[tw] OR ((singl*[tw] OR doubl*[tw] OR trebl*[tw] OR tripl*[tw]) AND (mask*[tw] OR blind*[tw])) OR “latin square”[tw] OR placebos [mh] OR placebo*[tw] OR random*[tw] OR research design [mh:noexp] OR comparative study [pt] OR evaluation studies [pt] OR follow-up studies [mh] OR prospective studies [mh] OR cross-over studies [mh] OR control [tw] OR controll*[tw] OR prospectiv*[tw] OR volunteer*[tw]) NOT (animals [mh] NOT humans [mh]))

OR

Systematic [sb].

The search terms above are specific to PubMed/MEDLINE and will not generalise to other search engines. Search strategies for the other databases were derived from the terms used in PubMed.

### Information sources and search strategy

With the assistance of a librarian, we designed a Boolean search strategy which includes variations in the term ‘palliative care’ and ‘intensive care’. We carried out the search strategy on the following databases: MEDLINE, EMBASE, Cochrane, CINAHL, and PsycINFO. The search terms that were used are outlined above. There were no date restrictions on the year of publications or on language. The preliminary peer review/scoping of the literature was conducted between 02 February 2018 and 19 May 2018. The formal systematic search of the literature was completed on 19 May 2018. The systematic search was conducted by a professional librarian (IvD) and assisted by three of the content experts (VM, DA, and SV).

### Inclusion and exclusion criteria

Inclusion criteria:

1. All adult patients (> 18 years of age) admitted to the intensive care unit
2. Palliative care intervention

Exclusion criteria include the following:

1. (Paediatric)  populations
2. Case series
3. Opinion paper/commentary
4. Review article (non-systematic review)
5. Lack of quantitative data
6. < 30 patients in study population
7. Conference abstracts/grey literature

### Study selection

After retrieving initial results, results were amalgamated and duplicates removed using RefWorks. The remaining studies were loaded onto the online systematic review software Covidence™. There was a total of five independent reviewers (VM, SV, DA, NA, and SB) who independently performed the title and abstract screening. Each title/abstract was reviewed independently by two reviewers. Where there was disagreement between reviewers, conflict was resolved between the two reviewers, erring on the side of inclusivity. A fifth author was consulted where consensus is not reached. Full texts will be retrieved for inclusion based upon the defined criteria. We will screen the references of included studies for potential publications not identified by the search strategy.

### Outcome measures

Our main objectives are to establish the number, type, and effectiveness of PC interventions in ICU, and to ascertain differences between Europe, North America, South America, and Asia. We will attempt to categorise outcomes of PC interventions according to previously established domains (patient- and family- centred care, communication, continuity of care, emotional and practical support of patients and families, symptom management and comfort care, spiritual support, and emotional and organisational support for ICU clinicians), to circumvent variations in terminology. We will also endeavour to assess the models for integrating palliative care into the ICU, identify research priorities, and formulate a research agenda to improve palliative care in ICUs.

### Data extraction

Data will be extracted onto a customised data extraction sheet by two independent reviewers. Conflicts will be resolved by discussion between the reviewing authors. A third author will be consulted where consensus is not reached.

Variables to be extracted will include but not be limited to

- Study data: year of data collection, year of publication, type of study, country of study, first author name, journal, total number of patients, clinical setting, and primary and secondary outcomes
- Patient population data: age, primary diagnosis, sex, life-limiting condition
- Palliative care intervention data: palliative care consults, end-of-life care, terminal care, advance care planning, living wills, ethics consultation, family meetings, and symptom management
- Outcome data: data including but not limited to: patient/family-, clinician-, system-, and content-related factors. These include patient- and family-centred care, communication, continuity of care, emotional and practical support of patients and families, symptom management and comfort care, spiritual support, and emotional and organisational support for ICU clinicians.

Where the study data is missing, we will attempt to contact the corresponding author.

### Evaluation of methodological quality of studies

Methodological quality of included randomised control trials will be assessed using Cochrane Collaboration’s tool for assessing risk of bias [13]. The following domains will be assessed for randomised controlled trials: random sequence generation, allocation concealment, blinding of sequence generation, allocation concealment, blinding of participants and personnel, blinding of outcome assessment, incomplete outcome data, selective reporting, and other bias. The risk of bias in each domain will be judged as either low, moderate, high, or unclear.

Methodological quality of included non-randomised trials will be assessed using the ROBINS-I tool. Seven domains of bias will be assessed including confounding, selection bias, bias in measurement classification of interventions, bias due to deviations from intended interventions, bias due to missing data, bias in measurement outcomes, and bias in selection of the reported result [14]. The risk of bias in each domain will be judged as either low, moderate, high, critical, or unclear.

To assess the risk of bias, we will rely on the information presented in the published literature. No reviewer will assess risk of bias for manuscripts they have participated in as an author.

### Analysis of outcomes

The initial analysis will be of a descriptive nature. The palliative care interventions and outcomes will be described. Subgroup analyses will include PC intervention incidence by region (Europe, North America, South America, and Asia). Where there is homogeneity between studies in the palliative care interventions used and measured outcomes, we will compare outcomes.

We will use the Mantel-Haenszel models for all dichotomous outcomes and we used the inverse variance method for the continuous outcomes. For continuous measures, we will calculate the mean differences. A random effects model will be used to analyse the data. Results will be presented as odds ratios with 95% confidence intervals for dichotomous outcomes. The meta-analysis will be carried out using review manager (‘Revman’) for Mac (version 5.1, Cochrane Collaboration, Oxford, UK). Statistical heterogeneity will be assessed using the *I*^2^ methodology. *I*^2^ values > 50% and > 75% are considered to indicate moderate and significant heterogeneity among studies, respectively. All *p* values will be two-tailed and considered statistically significant if < 0.05.

The strength of the body of evidence will be addressed using the GRADE (Grades of Recommendation Assessment, Development and Evaluation) system [15]. Any amendments to the protocol will be explicitly stated in the methods section of the final systematic review manuscript.

### Formulation of the recommendations

We will conduct a modified Delphi process to develop further recommendations for clinical practice and future clinical trials. The first step will consist of the working group developing draft recommendations; then, for the subsequent Delphi steps, we anticipate expanding to 20–25 members of the European Society of Intensive Care (ESICM) Ethics Committee with broad geographic (country) representation.

## Discussion

Patients admitted in ICU are often at high risk of dying and require invasive interventions or intensive monitoring. Among survivors, long-lasting sequelae affect patients’ quality of life, resulting in physical, cognitive and psychosocial complications [16]. Palliative care integrates the management of physical, psychological and spiritual symptoms, communication regarding goals of care, support for clinicians and families, and planning for care transitions in patients with a critical illness. PC is therefore an important component of ICU care for many patients, despite the traditional view that they constitute mutually exclusive forms of care. This view is more prevalent in Europe, where often the End-of-Life (EoL) needs of patients in ICU are addressed by intensivists, not palliative care specialists. The models for providing PC differ according to geographic and cultural criteria, rendering the comparison of outcomes problematic.

Integration of palliative care in ICU has been the topic of extensive research in the recent years. Previous systematic reviews have highlighted the heterogeneity of the PC interventions and the lack of consistent meaningful, multifaceted outcomes. We aim to bring together the evidence behind diverse interventions and outcomes of palliative care interventions in intensive care. This will facilitate the identification of key interventions which are consistently associated with positive clinical outcomes. Equally, we may be able to identify the lack of evidence behind specific interventions and outcomes that are potentially useful. This process will enable clinicians and researchers to identify the best interventions and outcomes for future clinical trials.

## Data Availability

Not applicable.

## Abbreviations

EoL: End-of-life
ESICM: European Society of Intensive Care
GRADE: Grades of Recommendation Assessment, Development and Evaluation
ICU: Intensive care unit
PC: Palliative care
PRISMA: Preferred Reporting Items of Systematic Reviews and Meta-Analyses
